# *ELMO2*-related intraosseous vascular malformation: new cases with novel pathogenic variants, clinical follow-up and therapeutic approaches

**DOI:** 10.1038/s41431-024-01739-z

**Published:** 2024-12-03

**Authors:** Mert Karakaya, Iman Ragab, Vera Riehmer, Florian Erger, Nihal Hussien Aly, Seung Woo Ryu, Go Hun Seo, Marc Hoemberg, Anne Maria Schultheis, Christian Netzer, Boris Decarolis

**Affiliations:** 1 Institute of Human Genetics, Faculty of Medicine and University Hospital Cologne, Cologne, Germany; 2 Center for Rare Diseases Cologne, Faculty of Medicine and University Hospital Cologne, Cologne, Germany; 3 Ain Shams University, Children’s Hospital, Pediatric Hospital, Hematology Oncology Unit, Cairo, Egypt; 4 Department of Pediatrics, IbnSina National College for Medical Sciences, Jeddah, Saudi Arabia; 5 Division of Medical Genetics, 3billion, Inc., Seoul, Korea; 6 Department of Pediatric Oncology, Faculty of Medicine and University Children’s Hospital of Cologne, Cologne, Germany; 7 Institute of Pathology, Faculty of Medicine and University Hospital Cologne, Cologne, Germany; 8 Department of Experimental Pediatric Oncology, Faculty of Medicine and University Children’s Hospital of Cologne, Cologne, Germany

**Keywords:** Tumour angiogenesis, Disease genetics

## Abstract

Primary intraosseous vascular malformation (VMPI, #606893) is an ultra-rare disorder caused by biallelic pathogenic variants in *ELMO2*. To date, only six families with pathogenic *ELMO2* variants causing a VMPI phenotype have been described. VMPI is characterized by vascular malformations that compress the facial bones, often leading to life-threatening complications, such as massive bleeding and intracranial herniation. In VMPI, vascular malformations are progressive and there is no causal therapy available. We report on four unreported individuals with classical VMPI harbouring biallelic truncating variants in *ELMO2*, including a novel homozygous 25 bp duplication c.579_603dup; p.(Leu202Profs*47), detected by whole-exome sequencing. We present extensive clinical follow-up data, including a close monitoring of an individual from prenatal diagnosis onwards. Using computed tomography or magnetic resonance imaging angiography, we described the radiological characteristics of vascular malformations with fast-flow properties in the affected individuals. Additionally, we conducted a comprehensive histopathological evaluation of samples from one individual. This analysis revealed not only the similar morphological features described previously but also some atypical findings, such as increased de novo bone formation. Furthermore, we report for the first time the use of propranolol and sirolimus in VMPI. While we noted a reduction of bleeding episodes in one individual, no significant clinical improvement was observed overall in the other individuals treated with sirolimus. Moreover, sirolimus led to severe infectious complications with abscess formation in two individuals. Conversely, propranolol was relatively well tolerated, although it did not result in any notable clinical outcomes. During follow-up, one individual died due to severe bleeding.

## Introduction

Congenital anomalies of vascular development are divided into two groups: Vascular tumors (mainly hemangiomas) and vascular malformations (VMs), which can be differentiated according to their histopathology, biological behaviour and clinical manifestation [1]. Primary intraosseous vascular malformation (VMPI, OMIM #606893, previously VMOS) is an ultra-rare disorder that represents the only inherited VM with autosomal recessive inheritance [2, 3]. In 2002, first familial cases of VMPI were identified in two families, in which patients exhibited severe intraosseous VM in craniofacial bones as well as midline abnormalities such as umbilical hernia and supraumbilical raphe [2]. Over a decade later, the molecular genetic cause of VMPI was identified by homozygosity mapping and exome sequencing, where biallelic germline loss-of-function (LoF) variants in *ELMO2* (Engulfment and cell motility gene 2, OMIM *606421) were identified in the two previously reported families and in three additional unrelated families [3].

The therapeutic approaches so far mainly included interventions such as sclerotherapeutic attempts and radical surgical procedures, e.g. full-mouth teeth extraction, calvarial bone excision and mandibulectomy. Pharmacotherapeutic attempts with interferon-α2a or radiotherapy did not significantly improve the course of the disease [3, 4]. Moreover, therapy is often complicated by life-threatening complications, such as increased intracranial pressure or recurrent gingival bleeding.

In this study, we report four previously unreported patients from three unrelated families with typical findings of VMPI. In addition, we present data on the clinical follow-up of affected individuals from prenatal diagnosis onwards and pharmacotherapeutic approaches to date for this very rare and fatal disease.

## Material and methods

### Study participants

**Fig. 1:**
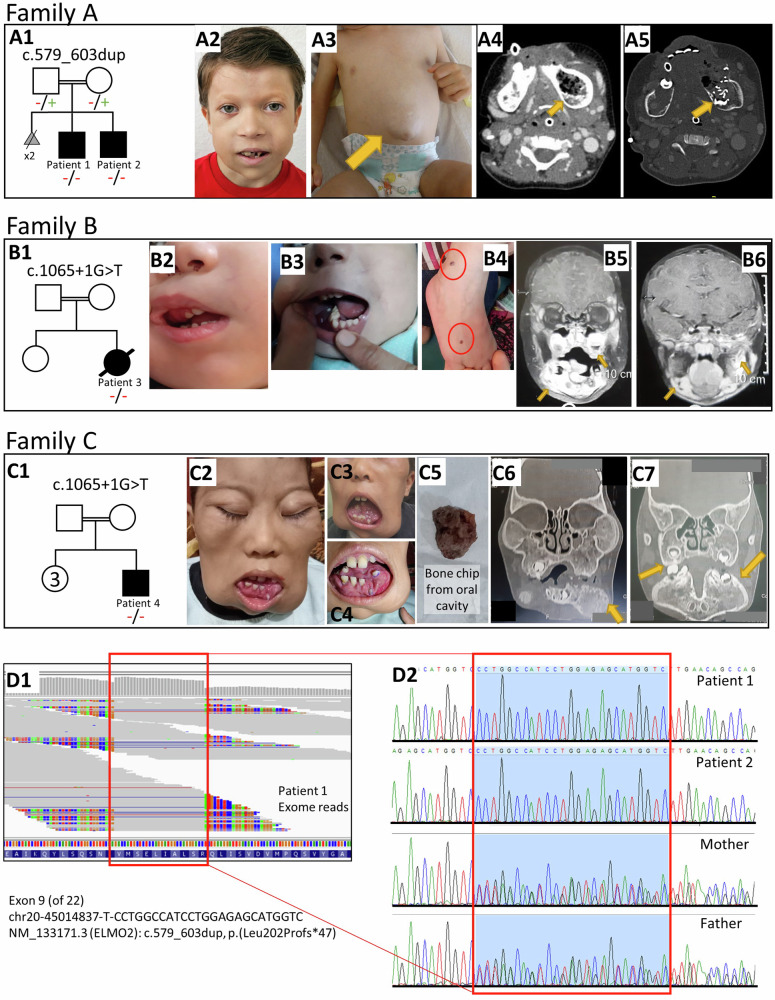
Clinical, Radiological, and Genetic features of affected individuals with ELMO2-related VMPI. (**A1**) Pedigree of Family A. (**A2**) Facial asymmetry with mandibular swelling and teeth deformity of patient 1 at 6 years of age. (**A3**) Umbilical hernia of patient 1 at 18 months of age. (**A4-A5**) CT with angiography reveals intraosseous expansion of hyperperfused blood vessels in left mandibula of patient 1. (**B1**) Pedigree of family B. (**B2-B3**) Facial asymmetry with expansion of right upper jaw with evidence of epistaxis and intraoral mucosal bleeding. (**B4**) Hemangioma-like skin lesions on right foot, 2-4 mm in diameter, marked in red circle. (**B5-B6**) Coronal T1 MRI images showing postcontrast enhancement of mandibular and maxillary lesions. (**C1**) Pedigree of family C. (**C2-4**) Marked expansion of facial bones with displaced teeth. (**C5**) Spontaneous detachment of a bone chip from oral cavity. (**C6-C7**) MRI images showing replacement of mandible and maxilla with small multilocular lesions encroaching on maxillary sinuses. (**D1**) Homozygote duplication of 25 bp visible based on the exome sequence reads of patient 1 generated from BAM data (Integrated Genomics Viewer, Massachusetts, USA). (**D2**) Sanger sequencing chromatograms confirm duplication as homozygote in affected brothers and as heterozygote in parents, Family A.

Proband 1 and 2 are the offspring of healthy and distantly related parents of Bulgarian origin (Fig. 1). Proband 3 is a female and proband 4 is a male affected individual, both from two unrelated Egyptian families from distant habitations with consanguineous parents (Fig. 1). Probands 1, 3, and 4 were referred to pediatric and genetic departments in order to elucidate the unusual presentation of craniofacial swellings and severe gingival bleedings. Proband 2 was diagnosed via prenatal testing upon the genetic diagnosis of his elder brother.

### Genetic analysis

Genomic DNA was extracted from whole blood samples of proband 1, 3 and 4 and their parents using standard techniques. The DNA of proband 2 was extracted from amniotic fluid sample at 18^th^ week of gestation. Whole exome sequencing (WES) was carried out on proband 1. Library preparation, sequencing and data analysis were performed as previously described [5]. Briefly, we used the Agilent SureSelect Human All Exon V7 target enrichment kit on an Illumina NextSeq 500 sequencer. Data analysis was done using an in inhouse-developed next-generation sequencing (NGS) pipeline. The sequenced reads were mapped to the reference genome (GRCh37/hg19), variant calling was done within the coding regions +/− 20 nucleotides of the adjacent non-coding DNA. Because of the highly specific clinical presentation, primary NGS data analysis was focused on the *ELMO2* gene. Sanger sequencing was performed to complete the segregation analysis in parental DNA samples and to perform the prenatal diagnosis for patient 2. For probands 3 and 4, WES was performed as previously reported [6].

### Pathological analysis of human tissue samples

The specimens of the two consecutive bone resections from proband 1 were used for comprehensive histopathological examinations. The sections were stained with hematoxylin and eosin (H&E). Immunohistochemistry was performed to characterize the blood vessels using markers such as CD31, D2-40, smooth muscle actin (SMA), and Desmin according to the protocols established for the routine clinical setting. Fluorescence In Situ Hybridisation (FISH) analysis was conducted to clarify a *USP6* translocation associated with aneurysmal bone cysts. Additionally, a comprehensive molecular pathologic assessment was performed using a massively parallel sequencing panel comprising fibrous dysplasia-associated genes (*CTNNB1, FRK, GNAS, HNF1A, IL6ST, JAK1*, and *STAT3*).

## Results

### Family A

**Fig. 2:**
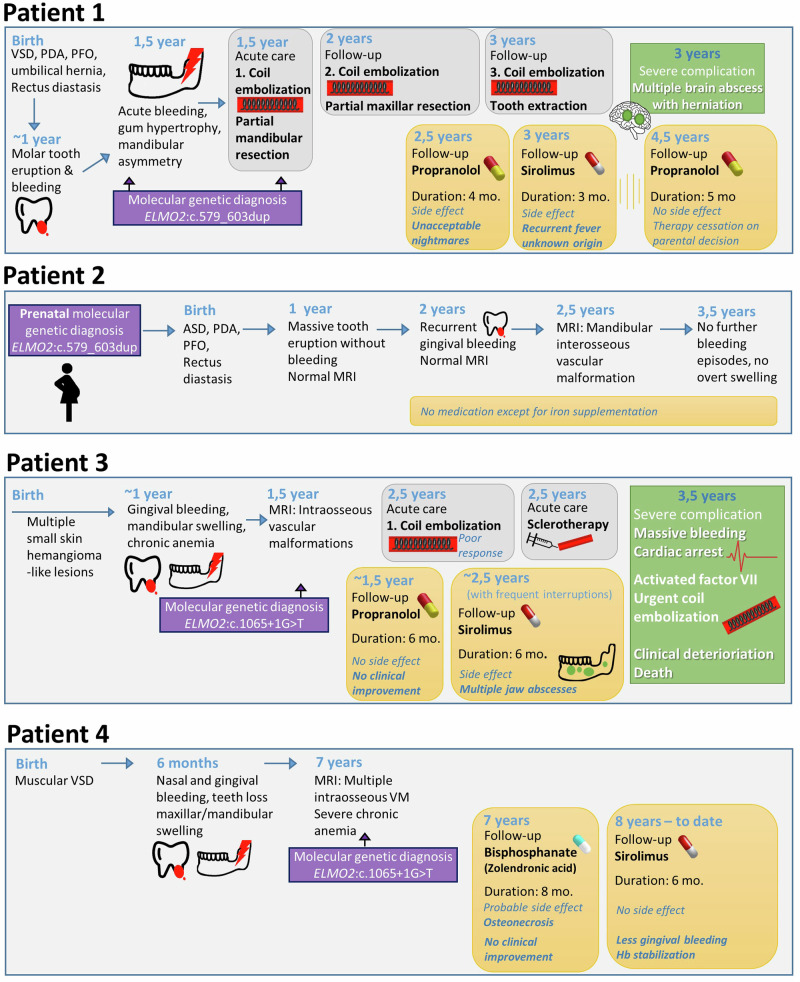
Overview and timeline of clinical symptoms, molecular genetic diagnosis and therapeutical approaches in affected individuals.

Proband 1 is currently 6 years old. He is the second child of healthy and distantly related parents of Bulgarian origin. The first two pregnancies were lost at later stages (35th and 25th gestational week) due to “cysts in brain” and cardiac abnormalities. At birth, he was diagnosed with a muscular type ventricular septal defect (VSD), a persistent ductus arteriosus (PDA), and a persistent foramen ovale (PFO) as well as an umbilical hernia with rectus diastasis but no specific intervention was required. At six months of age, the lower incisors erupted without any problems, whereas, the first molars erupted at around 11 months with remarkable swelling and gum bleeding. Subsequently, a significant gingival hypertrophy occurred and spread to the entire mandible. The rapidly progressive symptoms induced a facial asymmetry, recurrent mucositis, appetite loss and a failure to thrive (Fig. 1). At the age of 16 months, he was administered for a planned biopsy of the mandibular lesion during which massive bleeding occurred. To identify the cause of this unexpected, nearly-fatal bleeding from the mandibular region, a computed tomography angiography (CTA) was performed, which was compatible with an extensive hyperperfused arteriovenous malformation (AVM) originating from the lingual artery with hypertrophic arterial feeders. The feeder arteries were devascularized concurrently by coil embolization. The embolized region of the mandible was resected by excochleation on the following day (Fig. 2, Table 1).

**Table 1:** Clinical characteristics of affected individuals with VMPI.

	Family A		Family B	Family C
Clinical and genetic findings	Patient 1	Patient 2	Patient 3	Patient 4
Nucleotide change (NM_133171.3)	c.579_603dup	c.579_603dup	c.1065+1 G > T	c.1065+1 G > T
	homozygote	homozygote	homozygote	homozygote
Exon number	9	9	13	13
Predicted effect on protein	p.Leu202Profs*47	p.Leu202Profs*47	NP_573403.1:p.?	NP_573403.1:p.?
Sex	male	male	female	male
Ancestry	Bulgaria	Bulgaria	Egypt	Egypt
Current age	6 years	4 years	Died at 3,5 years of age	9 years
Age at first symptom	16 months	12 months	12 months	6 months
First symptom	Gingival bleeding	Massive tooth eruption	Gingival bleeding	Gingival bleeding
Course	Progressive	Slowly progressive	Progressive, death at 3,5 years of age	Progressive
Facial asymmetry	Yes	No	Yes	Yes
Exophthalmus	Yes	No	No	Yes
Swelling of facial bones	Yes	Yes	Yes	Yes
Ectopic tooth eruption	Yes	Yes	Yes	Yes
Heart defect	VSD, PFO, PDA	ASD, PFO, PDA	No	Muscular VSD
Midline anomalies	Diastasis recti, umbilical hernia	Diastasis recti	No	No
Extracranial bone involvement	No	No	No	No
Clinical complications				
Massive/acute bleeding	Yes – recurrent	Yes – single episode	Yes – recurrent	Yes – recurrent
Increased intracranial pressure	Yes (secondary to intracranial abscess)	No	No	No
Vision loss	No	No	No	No
Other	Multiple intracranial abscess	No	Skull bone involvement, multiple mandibular abscess, multiple skin hemangioma-like lesions	Skull bone involvement, separation of bony particles
Histopathological findings				
Tissue origin	Left mandibula	n.p.	n.p.	n.p.
Intraosseal vascular malformation	yes	n.p.	n.p.	n.p.
Fibrous dysplasia	no	n.p.	n.p.	n.p.
Radiological findings				
Imaging technique	CTA/MRA of facial bones, conventional angiography	MRA of facial bones	MRI skull bone with contrast/MRA/MRV	MRI skull bone
Native findings	VM with hyperperfusion in Mandibula and Maxilla	probable VM due to hyperintense signals in the zygomatic bone	Intramedullary tortuous vascular channels with enhancing vascular tufts prominent feeding vessels from branches of external carotid artery mainly the maxillary artery	Expansion of facial bones with displaced teeth. Multilocular maxillar and mandibular vascular lesions
Intervention	Coiling	n.p.	Coiling and sclerotherapy	n.p.
Recurrence of AVMs after intervention	Yes (new VMs in maxilla, mandibula, Os zygomaticum, Os sphenoidale)	n.p.	Yes	n.p.
Treatment				
Propranolol	Start: 2.5 years of age	Not received	Start: 1.5 year of age	Not received
	Duration: 4 months		Duration: 6 months	
	Discontinued due to intolerable nightmares		Discontinued at 2 years of age because of recurrent bleedings	
	Restart: 4.5 years of age			
	Duration: 5 months			
	Discontinued by parents’ decision			
Sirolimus	Start: 3 years of age	Not received	Start: 2.5 years of age	Start: 8 years of age
	Duration: 3 months		Duration: 6 months	Ongoing
	Discontinued due to recurrent fevers		Frequent interruptions due to mandibular abscess	Less frequent gingival bleeding
Bisphosphanate	Not received	Not received	Not received	Start: 7 years of age
				Duration: 8 months
				Discontinued due to no clinical response
Surgery (e.g. tooth extraction, mandibulectomy, reconstruction)	Partial mandibula resection, tooth extraction (64)	n.p.	n.p.	n.p.

*VSD* ventricular septal defect, *ASD* atrial septal defect, *PFO* patent foramen ovale, *PDA* patent ductus arteriosus, *AVM* arteriovenous malformations, *VM* vascular malformation, *CTA* computed tomography angiography, *MRA* magnetic resonance angiography, *MRV* magnetic resonance venography, *n.p.* not performed.

However, coil embolization had to be repeated twice in the maxillary branch arteries followed by a partial resection of the maxilla due to further bleeding episodes and formation of new VMs in zygomatic and sphenoid bones. At 2.5 years of age, empirical therapy with oral propranolol was started, which had to be discontinued after four months because of intolerable nightmares as a known side effect of the drug [7]. The pharmacotherapy was changed to sirolimus, but due to recurring fevers without identified focus and gingival bleeding episodes, the treatment had to be frequently interrupted and eventually discontinued after three months (Fig. 2). Control magnetic resonance imaging (MRI) after propranolol and during sirolimus therapy showed a mixed response with mild progression of some VM and slight regression of others. At three years of age, shortly after discontinuation of sirolimus, he presented with another episode of fever that was followed by neurological deterioration and signs of cerebral herniation. Emergency MRI revealed multiple brain abscesses which had to be drained surgically. *Streptococcus intermedius* were identified in cultures from the abscess. The exact cause of this severe infection remains unclear. However, a combination of invasion from a gingiva-dentogenic focus and sirolimus-related immunosuppression was assumed. Measurements of serum sirolimus concentration on different time points revealed drug levels in therapeutic range with trough levels between 6.7 and 8.6 ug/l. After this complication, the originally planned switch to everolimus as an alternative with a more favourable toxicity profile was abandoned. To exclude immunodeficiency, an immunological evaluation, especially of the neutrophilic function, was performed but revealed no signs of immunodeficiency. At 4.5 years of age, MRI showed another progression of the known VM. As surgical intervention—even after preparatory coil embolization—carried a very high risk of fatal bleeding, treatment with propranolol was resumed and has been well tolerated. However, the parents discontinued this treatment after five months as they felt that there was no change in bleeding frequency (Fig. 2). Currently, the proband only receives oral iron substitution to prevent iron deficiency due to chronic bleeding. The latest control MRI at 5.5 years of age showed a slow progression of the known mandibular and maxillary VM without any evidence of a novel intraosseous vascular lesion.

Upon diagnosis of proband 1, the family sought prenatal genetic counselling, as the mother was pregnant at that time, and opted for invasive genetic testing which revealed the diagnosis also in the fetus (see below ‘Genetic Findings’). The family decided to continue the pregnancy.

At birth, the newborn brother (proband 2, currently 4 years old) presented with a diastasis recti, an atrial septal defect, a PFO, and a PDA. First gingival bleeding occurred at the age of two. Cranial MRI at two years of age did not reveal any clear morphologic changes even though a potential small VM in the right zygomatic bone could not completely be excluded. The control MRI at 2.5 years of age revealed a distention of the mandible, suggestive of a potential interosseous VM. One year later, a new MRI showed no progression of the mandibular distention. So far, patient exhibited no further bleeding episodes and there is no overt clinical swelling of the upper nor lower jaw (Fig. 2, Table 1).

### Family B

Proband 3 from Family B was a girl of first cousin parents with Egyptian origin (Fig. 1). She had a history of four small (2-4 mm) hemangioma-like skin lesions, which appeared in the first year of life and became stationary (Fig. 1). At the age of one year, she presented with marked gingival bleeding and gradual swelling of maxilla (Fig. 1 and Table 1). She had developed a severe anemia with a drop of hemoglobin to 5 mg/dl and a transient isolated thrombocytopenia which resolved with intravenous immunoglobulin. A CT of facial bones revealed an expansion of skull base and facial bones with ground glass osteolytic foci. A dynamic MR-angiography (MRA) showed intraosseous expansion at maxillary bones and both parietal bones more on the left side, with intramedullary tortuous vascular channels, with enhancing vascular tufts at arterial phase in dynamic images, and with early venous filling and progressive enhancement in venous and delayed phase. MRA revealed feeding vessels from branches of external carotid artery, mainly the maxillary artery (Fig. 1). A treatment attempt with propranolol was initiated. However, after an initial improvement, daily gingival bleeding, epistaxis and progressive facial asymmetry with prominent right maxilla occurred. The therapy was switched to sirolimus at 30 months of age. During sirolimus therapy, she developed several dental abscesses, leading to frequent interruptions in therapy and making it difficult to evaluate the effectiveness of the therapy (Fig. 2). The mandibular abscesses were resolved with antibacterial treatment. The single blood culture done at one of the febrile episodes showed no bacterial growth. Sirolimus trough levels were between 4.3-5 ng/ml. Despite these complications, sirolimus was restarted to ensure stable drug levels and to evaluate the response to treatment. An embolization of the jaw lesion was unsuccessful and complicated by a severe bleeding, in which an intralesional sclerotherapy had to be performed to cease the bleeding. Another recent attack of massive gingival bleeding not responding to intravenous activated factor VII was complicated by severe acute decline in hemoglobin level and cardiac arrest. Upon reanimation, an urgent embolization of the internal maxillary had to be performed. However, she succumbed after the final severe bleeding attacks and could not be weaned from invasive ventilation. She passed away at the age of 3.5 years.

### Family C

Proband 4 from Family C is currently nine years old and the child of first cousin parents of Egyptian origin. Since the age of six months, he has been suffering from epistaxis and gingival bleeding with progressive mandibular, maxillary and calvarial swelling with bone and teeth loss (Fig. 1 and Table 1). CT and MRI of facial bones revealed hypertrophied facial bones with ground glass appearance. A therapeutic attempt with zolendronic acid was started because of the suspicion of fibrous dysplasia. However, the number of bleeding episodes and the size of lesions were increased and the patient lost a bone chip from oral cavity four months after the beginning of the bisphosphonate therapy. It is still unclear whether the observed complication arose from the primary disease-related bone degeneration or represents a therapy-related complication, specifically bisphosphonate-associated osteonecrosis of the jaw (BONJ). Given the relatively brief duration of bisphosphonate therapy preceding the occurrence (4 months), the likelihood of BONJ as the causative factor appears low, as the reported minimum time to jaw osteonecrosis with zolendronic acid injections is 10 months [8]. Ultimately, bisphosphonate therapy was discontinued after eight months due to poor response. The patient was started on sirolimus at eight years of age, which ameliorated the frequent gingival bleedings and stabilized haemoglobin levels without any recurrent fever or infectious complications, yet no regression in the size of lesions was observed.

#### Genetic findings

In proband 1, whole exome sequencing revealed a novel homozygous 25 bp duplication in *ELMO2* (NM_133171.3: c.579_603dup) resulting in a frameshift and a premature stop codon at amino acid position 248 (p.Leu202Profs*47) (Fig. 1). The variant was located in exon 9 (of 22) and has not been reported in the gnomAD database (v2.1.1 https://gnomad.broadinstitute.org/). It is predicted to cause nonsense-mediated decay, disrupt the Armadillo repeat 4 (ARM4) domain that mediates binding to RhoG and ILK proteins and to cause a truncation of the downstream essential domains. We classified the variant as pathogenic (class 5) according to the 2015 criteria of the American College of Medical Genetics [9]. Segregation analysis confirmed the heterozygous carrier status in the parents (Fig. 1).

The genetic diagnosis of proband 2 was made prenatally from genomic DNA isolated from amniotic fluid. The prenatal analysis of the fetal DNA via Sanger sequencing revealed the familial *ELMO2* variant in a homozygous state in the fetus (Fig. 1).

In proband 3, exome sequencing revealed a homozygous c.1065+1 G > T variant in *ELMO2*, affecting the previously reported canonical splice donor site [3], however with a previously undescribed nucleotide change (G > T instead of G > A).

In proband 4, exome sequencing revealed the same homozygous splice site variant as in patient 3 (c.1065+1 G > T).

#### Histopathological and molecular assessment

**Fig. 3:**
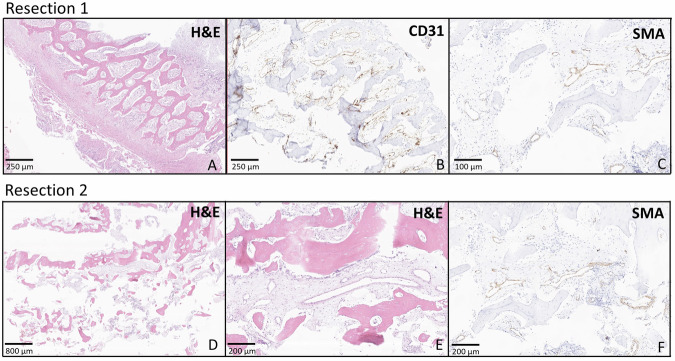
Histological and immunohistochemical findings. Panels **A**–**C** depict the specimen resected from mandibula in 2019 from patient 1. De novo bone formation in granulation-like tissue can be appreciated intermixed with small, thin-walled inconspicuous vessels (**A**). The endothelial cells in the vessel walls express CD31 (**B**) and have a retained pericyte layer expressing SMA (**C**), suggesting the presence of mature, capillary like vessels. Panels **D**, **E** show a specimen resected from maxilla in 2020 from the same patient. Irregular bone structures (**D, E**) with small vessels similar to the ones described above are seen. The vessels only have one layer of endothelial cells and a slim vessel wall with retained SMA-expressing pericytes (**F**). Both lesions give the impression of a reactive process rather than a tumor like lesion.

As proband 1 underwent three partial bone resections, two from the mandibular bone and one from the maxilla, we had the opportunity to study the histopathological presentation of the lesions. Evaluation of the beige-brownish, partly bone-hard mandibular tissue from the mandibular resections taken within a two months interval revealed a lesion composed of bone, fibrous stroma and numerous elongated, curvy, engorged blood vessels with erythrocyte extravasations. The stroma showed consistently focal reactive bone formation comprising irregularly shaped bone trabeculae of woven bone and thin-walled inconspicuous vessels (Fig. 3A, D, E). The thin-walled capillary vessels were positive for CD31 (Fig. 3B) and negative for D2-40 (not shown) and showed a retained expression of SMA (smooth muscle antibody) (Fig. 3C–F), but no expression of Desmin (not shown). The lesion did not fulfil criteria for arteriovenous malformation or any other neoplastic vascular process. However, it was noted that the vascular walls of the vessels exhibited dilatation and thinning. Histomorphologic findings were to some extend suggestive for an aneurysmal bone cyst (ABC) due to some pseudocystic areas and multinucleated giant cells. However, clinical presentation and radiological findings did not fit to an ABC and an *USP6* translocation that would further indicate an ABC was not identified using FISH analysis. We did not reveal any evidences of fibrous dysplasia on histomorphological assessment. Additionally, a comprehensive molecular pathologic assessment on the biopsy material, using a massively parallel sequencing panel comprising fibrous dysplasia-associated genes (*CTNNB1, FRK, GNAS, HNF1A, IL6ST, JAK1*, and *STAT3*) did not show any pathologic variant. Thus, as the genetic diagnosis of the patient had not been made at that point, the case was classified as most likely reactive tissue with a descriptive diagnosis of granulation tissue and reparative changes in the bone tissue. Re-evaluation with the knowledge of the genetic background did not substantially change this assessment because the presence of intact, mature, capillary like vessels not fulfilling criteria for arteriovenous malformation. Remarkable, however, was the abundant de novo bone formation in the overall granulation-like tissue which was present at both time points (Fig. 3).

## Discussion

VMPI (previously VMOS) is an ultra-rare disease that can result in death from life-threatening complications. The typical localization of vascular anomalies in craniofacial bones causing gingival hypertrophy and bleeding is indicative of this serious disease, which is caused by biallelic pathogenic variants in *ELMO2*. Early recognition of symptoms and timely definitive genetic diagnosis can enable education of the family and effective monitoring, which would reduce the severity of potentially lethal complications (e.g., massive bleeding or compression of vital organs). Furthermore, other preventive options such as preimplantation genetic diagnosis or prenatal diagnosis might be applicable in the presence of a molecular genetic diagnosis. Nevertheless, VMPI has a severe progressive course with high mortality rate. Among five previously identified individuals with long-term follow-up data, three patients died of disease-related complications in early adulthood at a mean age of 27 years [4]. In our cohort of four individuals, one individual (Proband 3) died from severe bleeding at the remarkably young age of 3.5 years, the youngest age of death reported in the literature.

Clinical presentation of probands in this study is similar to the already published patients emphasizing the severity of the clinical course also in younger children. Neither our four probands nor of the five cases originally reported showed any neurodevelopmental signs so far. However, two previous reports [10, 11] described *ELMO2* variants in the context of Ramon syndrome (OMIM #266270), as the probands in those studies exhibited seizures and intellectual disability (ID) as well as typical facial features compatible with fibrous dysplasia but also with VMPI [12, 13]. However, it cannot be determined from the existing data if the gingival lesions of the described individuals were compatible with fibrous dysplasia, which is a characteristic feature of Ramon syndrome [12]. At this time, it remains unclear if the neurodevelopmental features of those individuals from consanguineous families are caused by another unrevealed genetic variant or if the clinically distinct phenotype of Ramon syndrome may result from a distinct genotype-phenotype effect of the reported *ELMO2*-variants, as proposed by the authors [10, 11]. As two of our living patients are still very young, cognitive development of our patients will be monitored.

Uniquely, we are also closely monitoring one child (proband 2) who was diagnosed prenatally, allowing us to observe the disease’s progression from the very beginning, even before any symptoms appear. So far, his clinical course shows the development of mildly symptomatic VM with only one episode of mild bleeding. The optimal time to start a therapy is still unclear. It also remains uncertain which systemic treatment might improve the clinical course and prevent disease progression in affected individuals.

The loss of ELMO2 in patient-derived primary fibroblasts was found to be associated with a significant downregulation of the binding partner DOCK1, which impairs RAC1-dependent cell motility [3]. ELMO2 contributes in myoblast regeneration via DOCK1-interacting ELMO scaffold proteins to myoblast fusion. Whether it has a role in formation of vascular smooth muscles needs to be further studied [14]. However, the particular role of ELMO2 in the aforementioned pathways is yet unclear, which makes it challenging to predict the effect of the available drugs on the ELMO2 function.

Recent identification of two main signalling pathways in the pathogenesis of other VMs (PI3K/AKT/mTOR and RAS/RAF/MEK/ERK signalling) guided the implementation of promising agents in the therapy, such as mTOR-, PI3K-, AKT- and MEK-Inhibitors, thalidomide, and VEGF-Inhibitor (reviewed in ref. [15]). Pharmacotherapeutic agents acting upstream to the PI3K- and mTOR-pathways, such as bevacizumab (monoclonal VEGF inhibitor) and thalidomide, were shown to inhibit vascular proliferation significantly. Furthermore, they were shown to be efficient in the management of AVMs in clinical studies [16, 17]. However, studies on thalidomide were conducted exclusively in adult patients and there is no data regarding efficacy and safety in children or in patients with VMPI [17]. There is infrequent use of bevacizumab in children indicating a good safety profile [18, 19].

Our report is the first study in the literature on the use of sirolimus for VMPI. Sirolimus, also known as rapamycin, is an immunosuppressant drug from the class of mTOR-inhibitors, that was found to have anti-angiogenic and anti-proliferative effects. It has been shown to be efficacious and safe in complex vascular anomalies [20]. In our study, three probands (proband 1, 3, and 4) received an empirical therapy with sirolimus. However, the effect of this treatment could not be monitored adequately in two patients due to life debilitating side effects, such as recurrent fevers of unknown origin and abscesses in the brain and in mandible. Given this unusual side effect and the role of ELMO gene family in cell motility, we hypothesized that the cerebral abscesses could be related to dysfunction of the immune system. However, immunological assessment, performed in proband 1, revealed no pathologies.

Promisingly, in proband 4, we observed a cessation of severe bleedings and a normalisation of haemoglobin under sirolimus therapy at least as a short-term effect. Everolimus, a derivative of sirolimus with a more favourable toxicity profile, has also been shown to be effective in VM [21] and remains a potential alternative for further treatment. However, as it still has immunosuppressive effect, it was not used in our probands who experienced severe infectious complications during sirolimus therapy. Nevertheless, the role of sirolimus in these infections remains unclear, as the enlarged vessels that lead to recurrent bleeding also provide a possible gateway for bacteria.

Propranolol has been demonstrated to be an effective therapy for infantile hemangiomas by inhibiting the differentiation of hemangioma stem cells to hemangioma endothelial cells [22, 23]. Its efficacy on other vascular anomalies is however not proven and it is recommended to be used with caution [24]. Although we could only observe minimal effects of propranolol on the course of VMs in our probands, we intend to continue the propranolol use and monitor the long-term effects, as the drug has a good safety profile.

In our series, we identified a novel frameshift variant p.Leu202Profs*47 because of a 25 bp duplication in exon 9 of *ELMO2* (c.579_603dup) in Family A (proband 1 and 2). Until now, we observed a modest disease progress in the younger brother, who has been closely monitored since birth, in comparison to his older brother, who was diagnosed after a massive arterial bleeding. Further follow-up will determine if this novel duplication presents a phenotype with a clinical course different from that of previoulsy reported individuals.

In agreement with the previously described histopathological findings [2, 3], we have also observed aberrant thin-walled vascular channels in our histological assessment. However, both specimens of proband 1 which were taken at different time points and from different bones (mandible and maxilla) revealed normal CD31 and SMA expression, suggesting the presence of mature capillary-like vessels with no signs of abnormal architecture of the vascular wall. In both resected samples, we consistently identified a reactive process featuring a noticeably, unexplained de novo bone formation within granulation-like tissue and no overt evidence of a vascular malformation or any fibrous changes. It is also plausible that the reactive characteristics of the samples resulted from the inflammatory reaction to the prior surgical procedure and the severe bleeding that required immediate intervention. In summary, our observations reveal a more complex histopathological picture beyond simple vascular malformations. While ELMO2 deficiency has been linked to intraosseous vascular malformations, we would like to hypothesize that loss of ELMO2 functions might also cause a primary disorder of the craniofacial bones. This could also explain why the vascular abnormalities are restricted to intraosseous locations. However, since the data is still preliminary to draw a definitive conclusion, further research is needed to explore this hypothesis and elucidate the underlying mechanisms of ELMO2 deficiency and the downstream pathways, particularly RAC1 signalling, ultimately leading to a treatment approach based on a molecular mechanism of action.

In conclusion, the functional role of germline *ELMO2* mutations in the context of intraosseous VM is not yet understood, making it difficult to implement targeted therapeutic strategies for VMPI. We believe that molecular genetic diagnosis is a crucial first step in gaining more knowledge about the disease and its phenotypic expressions, which would also lead to the development of new therapeutic options. In our study, we report four additional clinical cases along with treatment attempts, histopathological review, and a novel pathogenic alteration. Combinatorial pharmacotherapies and innovative surgical procedures still need to be determined for the best possible care and treatment of patients with VMPI. To reduce the morbidity of the disease, iron supplementation could be considered in routine care.

## Data Availability

The datasets produced and/or examined in the present study can be obtained from the corresponding author upon a reasonable request.
